# Microbial Communities in Sunken Wood Are Structured by Wood-Boring Bivalves and Location in a Submarine Canyon

**DOI:** 10.1371/journal.pone.0096248

**Published:** 2014-05-07

**Authors:** Sonja K. Fagervold, Chiara Romano, Dimitri Kalenitchenko, Christian Borowski, Amandine Nunes-Jorge, Daniel Martin, Pierre E. Galand

**Affiliations:** 1 Sorbonne Universités, UPMC, Univ Paris 06, UMR8222, LECOB, Observatoire Océanologique, Banyuls-sur-Mer, France; 2 CNRS, UMR 8222, Laboratoire d'Écogéochimie des Environnements Benthiques (LECOB), Banyuls-sur-Mer, France; 3 Centre d'Estudis Avançats de Blanes (CEAB-CSIC), Blanes (Girona), Catalunya, Spain; 4 Max Planck Institute for Marine Microbiology, Bremen, Germany; University of Sydney, Australia

## Abstract

The cornerstones of sunken wood ecosystems are microorganisms involved in cellulose degradation. These can either be free-living microorganisms in the wood matrix or symbiotic bacteria associated with wood-boring bivalves such as emblematic species of *Xylophaga*, the most common deep-sea woodborer. Here we use experimentally submerged pine wood, placed in and outside the Mediterranean submarine Blanes Canyon, to compare the microbial communities on the wood, in fecal pellets of *Xylophaga* spp. and associated with the gills of these animals. Analyses based on tag pyrosequencing of the 16S rRNA bacterial gene showed that sunken wood contained three distinct microbial communities. Wood and pellet communities were different from each other suggesting that *Xylophaga* spp. create new microbial niches by excreting fecal pellets into their burrows. In turn, gills of *Xylophaga* spp. contain potential bacterial symbionts, as illustrated by the presence of sequences closely related to symbiotic bacteria found in other wood eating marine invertebrates. Finally, we found that sunken wood communities inside the canyon were different and more diverse than the ones outside the canyon. This finding extends to the microbial world the view that submarine canyons are sites of diverse marine life.

## Introduction

Debris of terrestrial plants can be exported from land to sea by rivers and streams, especially during flooding events [1]. Once saturated with water, the debris sinks and brings a discrete load of organic carbon to the ocean floor. The importance of plant debris for the oceans organic carbon cycle has recently been acknowledged [2]. However, sunken wood has long been in the center of scientific interest because it can harbor distinct and specialized faunal communities [3] and because sunken wood is hypothesized to play a key role in the maintenance and dispersion of chemosynthetic species in the deep sea [4], [5]. Among the emblematic species found on sunken wood are wood boring marine invertebrates, which use the wood matrix as shelter and food. A significant amount of energy is stored in the wood as cellulose which is degraded by cellulolytic organisms, and their degradation products can be used by animals that host microbial symbiotic communities [6]–[8].

*Xylophaga* Turton, 1822 from the family Pholadidae, is the most common genus of xylotrophic (wood eating) bivalves found in sunken wood in the deep sea [9], 10. These animals bore into wood using their shell and ingest the wood particles produced. They use wood as an energy source, but the origin of the cellulase used to hydrolyze the cellulose is still uncertain because studies on the physiology of *Xylophaga* do not exist. Shallow-water wood-boring bivalves from the family Teredinidae (known as shipworms) have been more extensively studied. Shipworms have strongly modified and enlarged gills that harbor cellulolytic and nitrogen-fixing *Gammaproteobacteria* [11], [12]. Cellulolytic enzymes produced by the gill endosymbionts are assumed to contribute to lignocellulose breakdown in the digestive system, however, this has not been demonstrated and the transfer mechanism of cellulases from the gills to the digestive tract remains unclear. The gills of *Xylophaga* are much smaller and less modified [13]. They also contain dense populations of endobacteria, but it is not known if these help digesting cellulose [14]. Further, one of the characteristics of *Xylophaga* species is that they fill their burrows with pellets of feces mixed with mucus while shipworms expel their fecal pellets outside the burrow. Thus, it is possible that *Xylophaga* create new niches for microbial colonization within the wood.

Microorganisms are also present in the wood itself where they degrade wood cells. As submerged woods become anaerobic after only a few days [15], it is expected that pathways associated to fermentation, with electron acceptors other than oxygen, play a significant role. Indeed, studies on sunken wood in the deep Mediterranean revealed that this substrate can harbor rich bacterial communities [16]–[18] including fermenting bacteria, microorganisms involved in sulfur cycling and methane production, and new clades of Bacteria and Archaea with unknown physiologies [18]. As the application of molecular techniques has revealed a large diversity of microbes associated to sunken woods, they have also allowed a first understanding of the ecology of sunken wood microbial communities, showing that wood type, immersion time and the environmental conditions surrounding submerged wood may promote contrasted bacterial communities [19], [16]–[18]. For instance, bacterial communities associated with oak wood that had been artificially submerged in the Blanes Canyon (western Mediterranean) were dominated by *Alphaproteobacteria* of the family *Rhodobacteriales*, *Gammaproteobacteria* and *Bacteroidetes* [17]. In contrast, bacterial communities recovered with wood deployments from the Eastern Mediterranean differed on higher taxon levels and they were characterized by the presence of *Flavobacteria* [16]. Moreover, factors controlling community assembly remain poorly understood and the possible effect of wood-boring bivalves on bacterial community composition has never been explored.

This study aims at investigating the composition and diversity of microbial communities in wood, and in particular if external factors exhibit structuring influence. A major focus is on the effects of wood-boring bivalves on the wood associated microbial communities, in particular with respect to the abundant fecal pellets deposited by the bivalves in their burrows. We hypothesize that fecal pellets increase habitat diversity and that pellet communities are distinct from those in the wood matrix. Our second goal was to test if the special environmental conditions inside submarine canyons provide structuring influence on the microbial wood communities, as canyons are known to be hotspots for benthic biomass and productivity [20]–[22]. We used pine wood that was experimentally deployed in the deep Blanes Canyon off the Mediterranean Spanish coast and in similar water depths on an adjacent open slope area. We characterized the microbial communities in the wood matrix and fecal pellets by analyzing the bacterial 16S rRNA gene targeted by 454 pyrosequencing. In addition, we analyzed bacteria in *Xylophaga* gills to test if they relate to wood communities.

## Materials and Methods

### Experimental set up

**Figure 1:**
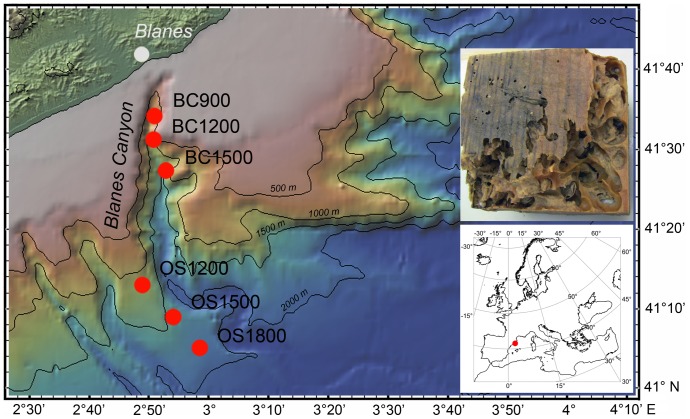
Map of sampling sites and wood with *Xylophaga* burrows. Map showing the position of the experimental moorings in relation to the Blanes Canyon (BC) and its outer slope (OS), drawn using GeoMapApp (http://www.geomapapp.org). Insert, cube of pine wood (8 X 8 X 8 cm) colonized by *Xylophaga* after 12 months of immersion in Blanes Canyon.

Traps with triplicate cubes (8×8×8 cm) of pine and triplicate cubes of oak wood were deployed along the axis of Blanes Canyon at 900, 1200 and 1500 m water depth. Additional traps of pine and oak cubes were deployed next to Blanes Canyon on the western outer slope at 1200, 1500 and 1800 m depth (Figure 1 and Table 1). The oak wood samples have been presented previously [17] but are included in Table 1 for a comprehensive overview. The traps were suspended 20 m above the seafloor. Cubes from 1200 m depth were collected in November 2009 after 9 months of immersion and samples from 900, 1500 and 1800 m depths were collected after 12 months of immersion. Pine cubes from Blanes Canyon and the outer slope were used for the analysis of microbial communities in the wood and in the fecal pellets of wood boring *Xylophaga* bivalves. Microbial wood communities in the oak cubes from Blanes Canyon have been analyzed previously but they were used here for comparative analyses of microbial fecal pellet communities in pine and oak, as the pellets in these oak tubes were not previously analyzed.

**Table 1:** Overview of the different samples in the whole experiment.

Trap	Depth (m)	Duration (Months)	Location	Samples	Wood type	Matrix
BC 900	894	12	Canyon	BC Pine 900 m	Pine	Wood
				BC Pine 900 m	Pine	Pellet
				BC Oak 900 m*	Oak	Wood
				BC Oak 900 m	Oak	Pellet
BC 1200	1195	9	Canyon	BC Pine 1200 m	Pine	Wood
				BC Pine 1200 m	Pine	Pellet
				BC Oak 1200 m*	Oak	Wood
				BC Oak 1200 m	Oak	Pellet
				Bla1.2	Pine	Gills
				Bla10	Pine	Gills
				Bla11	Pine	Gills
BC 1500	1468	12	Canyon	BC Pine 1500 m	Pine	Wood
				PC Pine 1500 m	Pine	Pellet
				BC Oak 1500 m*	Oak	Wood
				Bla1.1	Oak	Gills
OS 1200	1184	9	Slope	OS Pine 1200 m	Pine	Wood
				OS Oak 1200 m*	Oak	Wood
OS 1500	1497	12	Slope	OS Pine 1500 m	Pine	Wood
				OS Oak 1500 m*	Oak	Wood
OS 1800	1806	12	Slope	OS Pine 1800 m	Pine	Wood
				OS Oak 1800 m*	Oak	Wood

Details on the locations of the traps used for pine and oak (Fagervold et al 2013) wood immersion experiments, as well as the samples taken from each trap. Abbreviations Bla 1.2, Bla 10, Bla 11 and Bla 1.1 refer to gills extractions from four *Xylophaga* sp. A individuals.

* =  Samples from Fagervold et al (2013) that were also used in this study.

Immediately upon recovery of the cubes, wood chips to be used for microbial analysis were cut using sterilized tools, flash frozen in liquid nitrogen and kept at −20°C until further processing. Fecal pellets were collected from *Xylophaga* burrows in pine and oak cubes. Fecal pellets from individual wood triplicates were separately frozen at −20°C. Further, to estimate the % wood consumed, the rest of wood the cubes were carefully dissected by hand and all wood-boring bivalves were extracted and stored in 70% ethanol for taxonomic identification. To ensure that recently settled individuals were included, extraction was performed with the aid of a magnifier (2X) or a dissecting microscope. Shell length (SL) of each specimen of *Xylophaga* spp. was measured to the nearest 0.1 mm with digital calipers. The volume of each *Xylophaga* was calculated as the volume of a sphere with a radius equal to SL/2. Considering that each specimen created a burrow in the wood, the volume consumed by each *Xylophaga* was estimated to three time its volume. The % of wood consumed was estimated as the ratio between the volume occupied by *Xylophaga* spp. and the total volume of the wood cube.

No specific permissions were required for deploying submerged moorings in Blanes Canyon as it is not a protected area, moreover this study did not involve endangered or protected species.

### Wood and pellet DNA extraction, PCR and pyrosequencing

Procedures for DNA extraction, PCR and pyrosequencing were performed as described earlier [17]. Briefly, representative pieces from each wood cube used for analyses of microbial wood communities (Table 1) were powdered by bead beating (RETSCH Mixer Mill. Retsch, Inc. MM 301) using 25-ml grinder jars (Retsch, Inc. MM 400 Stainless steel) and 20-mm diameter stainless steel balls. The grinder jars were dipped into liquid nitrogen to keep the wood brittle. The fecal pellet material was already in powder form after drying. Approximately 100 mg of the powders from woods or fecal pellets were used to extract genomic DNA with the Mobio PowerPlant kit (Ozyme, Saint-Quentin-en-Yvelines, France).

Initially, DNA extracts from individual triplicates of two selected treatments were amplified and sequenced: 1200 m pine wood chips and 1200 m pine pellet. Bacterial communities were then compared in a cluster analysis (see below), together with communities from oak wood obtained from a previous study from the Blanes Canyon [17]. The results revealed that community composition of individual triplicates were always more similar within than across treatments (Figure S1). We therefore pooled the triplicate DNA extracts from each treatment for further processing. This resulted in 6 pooled DNA extracts for wood (3 pine from the Blanes Canyon and 3 from the outer slope) and 5 pooled DNA extracts from fecal pellets (3 from pine and 2 from oak inside the Blanes Canyon) (Table 1).

A portion of the 16S rRNA gene was amplified by PCR using modified versions of universal bacterial 16S rRNA primers 27F (5'-AGRGTTTGATCMTGGCTCAG-3') [23] and 519R (5'-GTVTTACCGCGGCTGCTG-3') [24] as described in previously [17]. Amplicons obtained with the 27F primer were modified at the 5′ end by addition of the Roche 454 A-adaptor sequence and a 10-nucleotide identifier barcode (multiplex identifier, MID). Emulsion PCR and Roche 454 pyrosequencing (Genome Sequencer, FLX Titanium chemistry) were performed at the Genotoul platform of INRA, Toulouse (France).

### Dissection, DNA extraction, PCR and pyrosequencing of *Xylophaga* gills

Bacterial 16S rRNA gene sequences from *Xylophaga* gill bacteria originated from a separate study using a different sequencing approach. Four individuals of *Xylophaga* sp. A, which was the most abundant wood-boring bivalve in the Blanes Canyon pine and oak deployments [25] were dissected. Three individuals originated from pine (Bla1.2, Bla10, Bla11), one from oak (Bla1.1; Table 1). DNA was extracted from their symbiont-containing gills following the protocol of Zhou et al. [26]. Amplification and 454 pyrosequencing of ∼480 bp long DNA fragments covering the V3 region of bacterial 16S rRNA genes was performed using primers bac339F (5′-CTCCTACGGGAGGCAGCAG-3′) and bac815R (5′-TTGTGCGGGCCCCCGTCAATT-3′) in a commercial laboratory (MR DNA, Shallowater, TX, USA). Bacterial DNA was amplified in a single-step PCR in which adaptors and barcodes were linked to the 5' region of the amplicons using the HotStarTaq Plus Master Mix Kit (Qiagen, Valencia, CA, USA). Amplification conditions were as follows: 94°C for 3 min, 28 cycles at 94°C for 30 s, 53°C for 40 s, 72°C for 1 min, and a final elongation step at 72°C. Equal concentrations of all PCR products were combined and purified with Agencourt Ampure beads (Agencourt Bioscience Corporation, MA, USA). Combined samples were sequenced with a Roche 454 FLX titanium instrument and reagents, following the manufacturer's procedures.

Attempts to amplify bacterial 16S rRNA genes from dissected gut tissue with the general bacterial primers 8F and 1492R [27] were unsuccessful and analysis of microbial gut communities was not further followed.

### Sequence data analyses

All reads that had mismatches to the 16S rRNA primers, contained ambiguous nucleotides (N) or were shorter than 270 nucleotides (excl. the forward primer) were removed. The remaining sequences were subjected to stringent quality trimming to remove reads containing ≥3% bases with Phred values <27 (0.2% per-base error probability). This minimizes the influence of erroneous reads when clustering at 97% for OTU definition [28], [29]. Sequences were then de-replicated and clustered at a 97% threshold using Uclust [30]. Sequences from each OTU were classified by comparison to the Greengenes database [31]. Read quality filtering and length trimming, dereplication, clustering at 97% sequence identity, taxonomic classification and dataset partitioning based on barcodes were conducted with Pyrotagger [32]. The taxonomic affiliations of the most abundant OTUs (>1% of the sequences) were further verified against sequences from the NCBI databases using BLAST [33]. To compare bacterial communities for diversity analysis, all samples were randomly resampled to the size of the sample containing the fewest sequences (n = 798) using Daisy Chopper [34]. Calculation of the Shannon diversity index (H′) and cluster analysis were performed using the software PAST [35]. A similarity percentage analysis, SIMPER [36], was conducted to identify the phylotypes contributing the most to the dissimilarity between different samples. Sequences have been submitted to MG-RAST (http://metagenomics.anl.gov/linkin.cgi?project=5773) for public availability.

Because the 16S rRNA genes of free-living bacteria and gill bacteria from *Xylophaga* were amplified with different primer pairs, the obtained sequences did not cover identical gene fragments. However, the overlap included the entire hypervariable V3 region (*E. coli* positions 433–497) that is widely used in phylogenetic studies using next generation sequencing methods, and this region yields sufficient information for a direct comparison between wood, pellet and gill communities of *Xylophaga*. All sequences were therefore realigned and an OTU table based upon 100% sequence identity built from the common overlapping region of 80 bp. The analysis was done in mothur [37] using the Silva SEED database provided as a reference alignment.

### Network association

A network analysis was conducted to characterize the relationships among bacterial OTUs as described earlier [38]. Maximal information-based nonparametric (MINE) statistics were applied by computing the maximal information coefficient (MIC) between each pair of OTUs [39]. MIC captures associations between data and provides a score that represents the strength of a relationship between data pairs. A matrix of MIC values >0.5 and corresponding to positive linear correlations was used with Cytoscape 2.8.3 to visualize the network of associations [40]. In these visualizations, bacterial OTUs are represented as nodes and are connected by lines that are proportional in length to the MIC value. The force-directed layout based on the Fruchterman-Rheingold Algorithm [41] was edge-weighted by the MIC value.

## Results

### Wood degradation and bacterial diversity

Pine wood showed different levels of degradation depending of the location, wood loss being higher in BC than in OS (18.3 vs 2.8% in average, respectively) (Figure S2). *Xylophaga* spp. had degraded large inner parts of the cubes, leaving their burrows filled with wood pellets (Figure 1, insert). Sequencing of the bacterial 16S rRNA gene originating from wood and pellet yielded a total of 55 874 quality checked sequences. The amount of sequences varied among samples but rarefaction analysis (Figure S3) shows that the sequencing effort was not exhaustive. Bacterial community diversity was significantly higher in the wood immerged in Blanes Canyon compared to wood deployed on the open slope (Figure S3), as estimated with the number of OTUs and the Shannon index (t-test, p = 0.01, Table 2). On the other hand, the bacterial diversity found in the pellets filling the burrows of *Xylophaga* spp. from the canyon did not differ significantly from that in the wood matrix (p = 0.09).

**Table 2:** Alpha diversity.

		Subsampled (n = 798)			
	Sample	OTUs	H′	Chao-1	Cov
Wood	OS Pine 1200 m	169	4.37	259	91
	OS Pine 1500 m	166	4.06	284	90
	OS Pine 1800 m	174	4.46	263	91
	BC Pine 1200 m	229	4.81	399	85
	BC Pine 1500 m	251	4.93	434	83
	BC Pine 900 m	244	4.85	456	83
Pellet	Pine 1200 m	202	4.45	303	89
	Pine 1500 m	142	3.84	192	93
	Pine 900 m	235	4.65	491	83
	Oak 900 m	194	4.51	286	89
	Oak 1200 m	127	4.13	156	95
Gills	Bla1.1	131	3.81	313	91
	Bla10	66	1.69	86	97
	Bla11	39	2.58	41	99
	Bla1.2*	7	0.57	7	99

Number of sequences and diversity of wood, pellet and gill samples from Blanes Canyon (BC) and its open slope (OS). H′: Shannon index, Chao-1: Chao true diversity estimator, Cov: coverage.

*not subsampled (n = 263).

### Bacterial community composition in the wood

**Figure 2:**
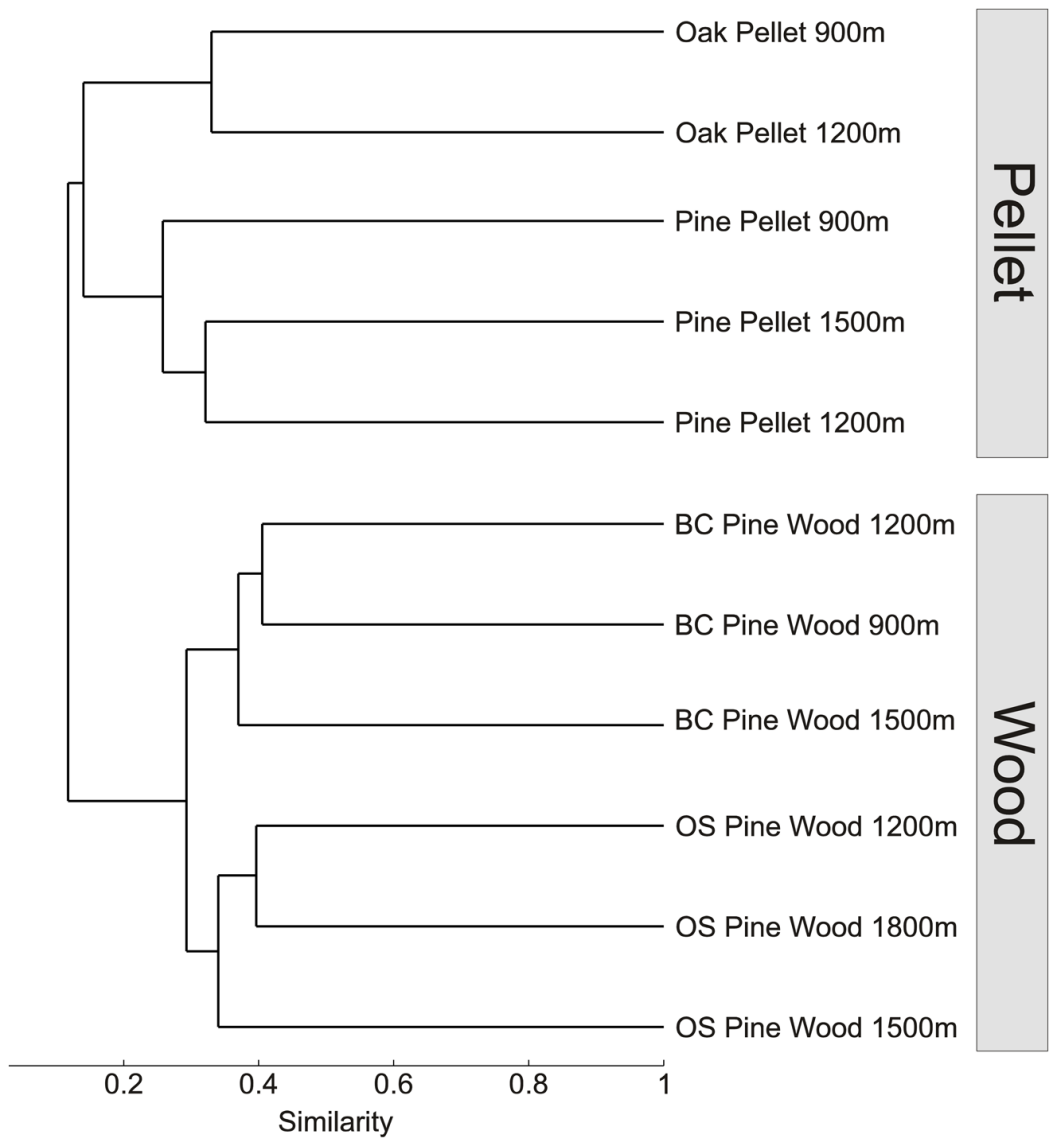
Sample clustering. Dendrogram based on Bray-Curtis distance representing the similarity between bacterial communities sequenced from the wood matrix and from burrow pellets obtained from wood immerged at various depths in Blanes Canyon (BC) and its adjacent open slope (OS).

Cluster analysis at the OTU level based on a Bray Curtis distance matrix showed that bacterial communities could be separated in two main groups: pellet and wood communities (Figure 2). Within the wood samples, communities grouped according to location. All samples from Blanes Canyon grouped together and were separate from the open slope samples. Further, a comparison of sequences from this study with oak wood sequences from Fagervold et al. [17] showed that pine wood communities were different from oak wood communities (Figure S1). This was also true when comparing the pellet samples only, in that they grouped according to wood type. Depth was not a structuring factor for the community composition in pellets (Figure 2).

**Figure 3:**
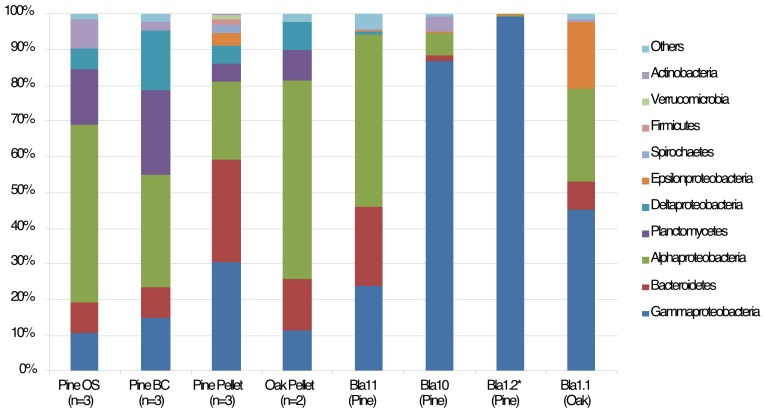
Distribution of bacterial taxa. Relative proportion of the most abundant bacterial phylum or class (>1% of the sequences) found in wood, pellet and gill (Bla) samples from Blanes Canyon (BC) and its adjacent open slope (OS). *  =  contained significantly less sequences than the rest of the samples (n = 263).

The microbial composition differed at high taxonomic level (phylum/class level) (Figure 3). Pine wood samples contained more *Alphaproteobacteria*, *Planctomycetes* and *Deltaproteobacteria* while pine pellet communities contained more *Gammaproteobacteria* and *Bacteroidetes* (Figure 3). Regarding wood samples, canyon communities harbored less *Alphaproteobacteria* and more *Deltaproteobacteria* than the open slope. Concerning pellet samples, oak contained more *Alphaproteobacteria* than pine. In turn, gill communities were dominated by *Gammaproteobacteria* sequences with the exception of the sample collected from oak, Bla11, which contained more *Alphaproteobacteria* (Figure 3).

### OTU co-occurrence and taxonomy in the wood

**Figure 4:**
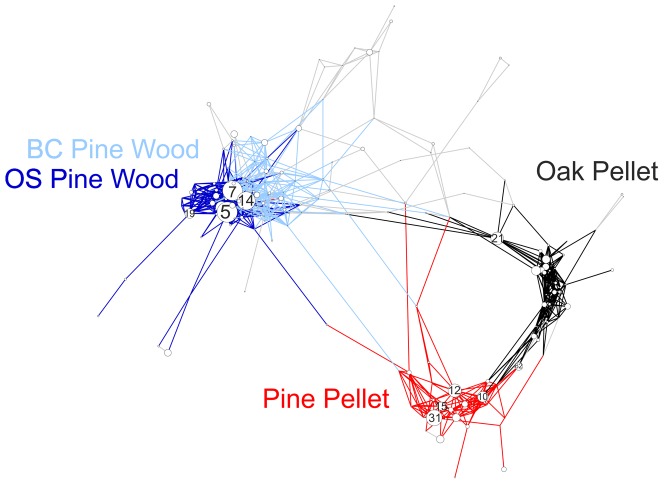
OTU network. Network showing associations between OTUs found in wood and pellets from wood immerged at various depths in Blanes Canyon (BC) and its adjacent open slope (OS). OTUs are represented as nodes (white circles) in the network and the size of each node is proportional to the number of sequences contained in each OTU. Lines connect the nodes that are the most correlated (MIC values>0.5). The identifying numbers of the most abundant OTUs are written in the nodes. Colors highlight associations between the OTUs that most explain the differences between groups of samples (Oak pellet, Pine pellet, BC Pine wood and OS Pine wood). OTUs that best explain differences between samples were identified by a SIMPER analysis.

Microbial communities in pine pellets from Blanes Canyon shared very few OTUs with wood samples from the same wood cubes, and the few OTUs in common were not abundant (Figure 4). Among the typical pine pellet OTUs, the most abundant were OTU 31, a *Gammaproteobacterium* distantly related to unpublished sequences from a marine biofilm, followed by OTU 10, a *Bacteroidetes* distantly related (90%) to algae associated communities, a *Gammaproteobacterium* (OTU 12) distantly related (93%) to sequence from sunken wood [42], and an *Epsilonproteobacterium* (OTU 15) previously detected as abundant in sequences cloned from pine pellets (OTU6, [19]). Among less abundant pellet OTUs, one *Gammaproteobacterium* (OTU 83) exhibited 99% similarity to a symbiotic bacterium from the Teredinidae *Lyrodus pedicellatus* [43].

Pine pellet communities had more in common with those from oak pellets than from the pine wood. Nevertheless, the oak pellets contained more *Alphaproteobacteria* closely related to coral tissue clones [44], [45] (OTU 21) (97%). Among the OTU shared between pellet samples, many belonged to *Bacteroidetes* often distantly related to sponge bacteria (OTU 62 and 77, 94–95% similarity, [46] or deep sediments (OTU 45, 95% similarity, [47]).

On the other hand, communities from wood in the canyon shared many OTUs with open slope communities, as illustrated by the short distance separating these samples in the network (Figure 4). In pine wood from the canyon, an abundant *Planctomycetes* OTU (OTU 14) was 100% similar to sequences found in the digestive tract of a chiton, a wood associated mollusk [42], and it was 99% identical to digestive tract bacteria found in a sea urchin from a wood fall [48]. Further, an abundant *Deltaproteobacterium* OTU (OTU 51) was closely related (99%) to clones found in sunken wood [42]. A less abundant *Gammaproteobacterium* was distantly related to *Teredinibacter turnerae*, an intracellular endosymbiont of shallow water marine wood-boring Teredinidae [49]. In wood from the open slope, the most abundant OTU (OTU 5) belonged to the *Rhodobacteriales* order of *Alphaproteobacteria* and was 100% similar to a sequence found earlier in oak wood from Blanes Canyon (Blanes 1043, [17]) and distantly related (91%) to bacteria found in guts of the marine wood-feeding gastropod *Pectinodonta* sp. (Patellogastropoda, Mollusca) [50] and the chiton *Nierstraszella lineata* [42]. Another abundant OTU (OTU 7) was identified as a *Gammaproteobacterium* related to clones from gorgon or seafloor lava (96%). This OTU was not detected in previous oak wood samples from Blanes Canyon.

### *Xylophaga* gill community

**Figure 5:**
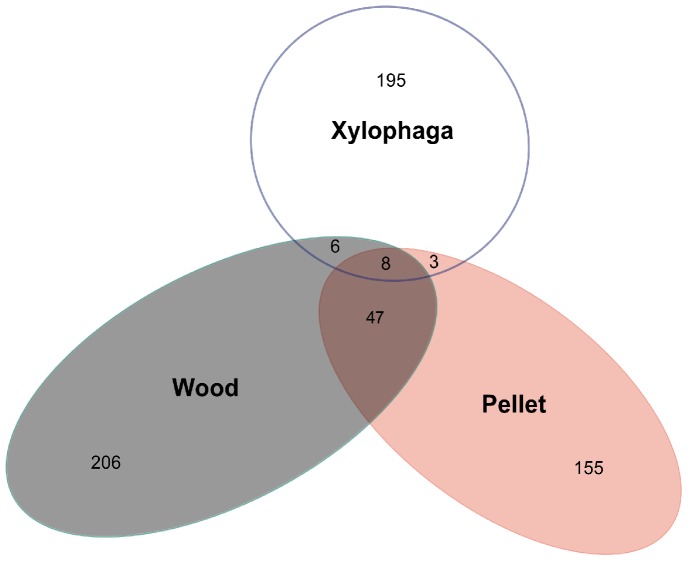
Venn diagram representing bacterial OTUs unique and common to the wood samples, the pellet and the *Xylophaga* gills.

A total of 8260 sequences were obtained after quality check from the four individuals of *Xylophaga* (Table 1). The number of sequences varied between samples as did the diversity, but the gill community from *Xylophaga* was always less diverse than wood or pellet communities (Table 1). Bacteria from gills of *Xylophaga* were different from those found in wood and pellets (Figure 5). Only 17 *Xylophaga* OTUs (8%) were found in the pellets or in wood while pellets and wood shared 55 of their OTUs (21 to 26%) (Figure 5).

Many sequences from the gills of *Xylophaga* were closely related to those of symbiotic bacteria from shipworms. In specimen Bla1.2, at least 96% of the sequences were from possible symbionts. The most abundant OTU, which represented 84% of the sequences, was 99% similar to a sequence of a *Gammaproteobacterium* from the teredinid shipworm *L. pedicellatus* (Clone RT14, [43]). In Bla10, at least 75% of the sequences were from possible symbionts. The most abundant OTU (73%) was 95% similar to the 16S rRNA gene of the T7901 strain of the Gammaproteobacterium *T. turnerae* [49]. In Bla11, more than 45% of the sequences were possible symbionts. The most abundant OTUs were less dominant than in the other *Xylophaga* specimens with only 24% of the sequences distantly affiliated to a symbiont of *L. pedicellatus* (Clone RT20, [43]). For specimen Bla1.1, we probably failed to amplify specific gill symbionts as the most abundant OTU related to symbionts from Teredinidae represented only 5% of the sequences.

## Discussion

The incubation of wood pieces in Blanes Canyon and its adjacent open slope showed that the boring activity of *Xylophaga* sp. A transformed the wood environment by creating distinct niches for bacterial communities. The communities associated with the gills of *Xylophaga* were different and probably composed of symbiotic bacteria, while the niche created by the pellets promoted the development of bacterial communities that were very different from those in the wood matrix. The large differences observed at phylum and OTU levels suggest that the pellet populations represent a distinct community rather than a subset of the wood communities.

The accumulation of pellets consolidated with mucus is typical for *Xylophaga* [13]. While Teredinidae expulse the products of their wood boring activity from their burrows, *Xylophaga* species most often form chimneys of compacted fecal pellets around their siphons, thus lining the bored tunnels. These pellets have a very different chemical composition compared to wood, as up to 80% of the wood cellulose may be lost during digestion by *Xylophaga* spp. [51]. Pellets may thus form a new substrate for microorganisms that is depleted in cellulose but enriched in mucus, in comparison to wood. Alternatively, the community found in pellets might be similar to that found inside the gut of *Xylophaga*. These animals ingest the wood shavings produced by their shell, which they use as rasp to dig burrows. Wood particles are then stored in a large caecum before passing through the stomach and intestine. However, previous studies found few microbes in the caecum of *Xylophaga* [14] and Teredinidae [52] while large numbers of bacteria occurred in the fecal pellets in the intestines of Teredinidae [52]. It is not known if bacterial communities follow the same spatial distribution in the digestive tracts of *Xylophaga*, but similar life strategies and common evolutionary history of the two wood-boring bivalve groups [53] may suggest so. Bacteria colonizing the pellets may thus exit the digestive tract with the excreted feces and survive in the burrows. However this hypothesis could not be verified in this study, as the gut microbiota was not analyzed. Further, communities from pine pellets were different from those in oak pellets. This difference could be due to the presence of a second species, *Xylophaga* sp. B, that was predominantly colonizing pine while it was rare in oak [25]. It is possible that this species has different gut microflora than the ubiquitously abundant *Xylophaga* sp. A [25] and this could influence the composition of pellet communities in pine and oak. However, it is also possible that the composition of the wood itself is controlling the composition of pellet communities. Remarkably, we could not identify known cellulolytic strains among the bacteria detected in the fecal pellets. However, since cellulotyic pathways are widespread over many bacterial phyla, it cannot be excluded that the uncultured bacteria we detected may be able to degrade cellulose. One metabolic pathway that might be inferred from the identified OTUs is fermentation. OTU 17 shares 98% sequence identity to the fermentative Alphaproteobacterium *Polymorphum gilvum* [54], and this close relationship may be an indication that fermentative bacteria can take advantage of the wood remains.

We detected several abundant OTUs in the gills of *Xylophaga* sp. A closely related to sequences from bacteria associated to wood-boring Teredinidae. The very close similarity (99%) of these OTUs to symbionts from the shipworm *Lyrodus pedicellatus* is a strong indication that we were able to detect gill symbionts from *Xylophaga* sp. A. Bacteria had been observed earlier in gills of *Xylophaga* [14] but they have never been isolated or taxonomically characterized. Further, the most abundant OTU in *Xylophaga* sp. A matched the symbionts of the most abundant phylotype (clade P3) in the Teredinidae *L. pedicellatus* [43]. We also detected sequences related to the cultivated shipworm endosymbiont *Teredinibacter turnerae*, a cellulolytic *Gammaproteobacterium* that has been isolated from many teredinid host species [12]. The presence of similar sequences in deep-sea and shallow environments indicates that wood-boring endosymbionts may be adapted to a wide range of environments and hosts.

The location of the sunken wood, a factor that is linked to different levels of wood degradation, clearly shaped the community composition in the wood matrix. Wood deposited in the canyon, which was largely and more rapidly degraded and colonized by the wood-boring bivalves, harbored different and more diverse communities than wood in the open slope. Canyons channel the transport of organic matter from the continental shelf to the deep sea, which is specially enhanced during dense shelf water cascading events [55]. This process in combination with frequent storms from easterly directions in the western Mediterranean area and offshore convection appear to be the main drivers for the transfer of organic matter to the deep Mediterranean Sea [56], [57] and they may in particular contribute to high benthic biomass and productivity that make the hot spot character of submarine canyon ecosystems [21]. Our results thus extend to the microbial world the view that submarine canyons are sites of enhanced marine diversity. The difference in bacterial community composition between woods immerged inside and outside the canyon may also be due to different sources of microbes colonizing that wood. Allochthonous bacteria could be transported to the canyon as a result of ecosystem forcing events, which may lead to the establishment of different communities in the canyon water column or sediments compared to the open slope. However, we cannot discard the possibility that the difference in bacterial communities between locations are linked to the different phases of the wood decomposition process. The pine wood communities where characterized by a large proportion of *Alphaproteobacteria* followed by *Planctomycetes* and *Gammaproteobacteria*. These classes of bacteria were also observed previously in oak wood [17], emphasizing their role in wood degradation. However, there were differences between pine and oak at the OTU level. The most abundant pine OTUs were absent in oak, like the *Gammaproteobacterium* OTU 7, or less abundant, as in the case of the *Alphaproteobacterium* OTU 5. Some pine OTUs were also 100% similar to sequences found on sunken wood, inside a chiton gut [42], and in a sea urchin collected around the Vanuatu Island [48]. This suggests that some bacteria associated with sunken wood are distributed worldwide. The presence of these bacteria in the guts of different wood-ingesting animal species suggests that they may either be associated to a large variety of macroorganisms or that they may represent globally distributed sunken-wood microorganisms that are ingested by the animals together with the wood.

## Supporting Information

Figure S1 **Clustering of all samples.** Hierarchical clustering using the Bray Curtis index showing the similarity of the microbial communities between the different samples.
(PDF)

Figure S2 **Wood consumtion.** Percentage of consumed pine wood after immersion at various depths in Blanes Canyon (BC) and its adjacent open slope (OS), expressed as percentage of the initial volume of the respective wood cubes.
(PDF)

Figure S3 **Rarefaction curve.** Number of sequences versus OTUs formed for all sequenced samples, except the gill samples. Broadly, blue lines represents BC Pine Wood, redish lines OS Pine Wood, greenish lines Pine Pellet sampels and purple lines oak pellets.
(PDF)
