# Supplementary figures and images for: Microbial Communities in Sunken Wood Are Structured by Wood-Boring Bivalves and Location in a Submarine Canyon
Source: PLoS One. 2014 May 7;9(5):e96248. doi: 10.1371/journal.pone.0096248 (PMC4013006; doi:10.1371/journal.pone.0096248)

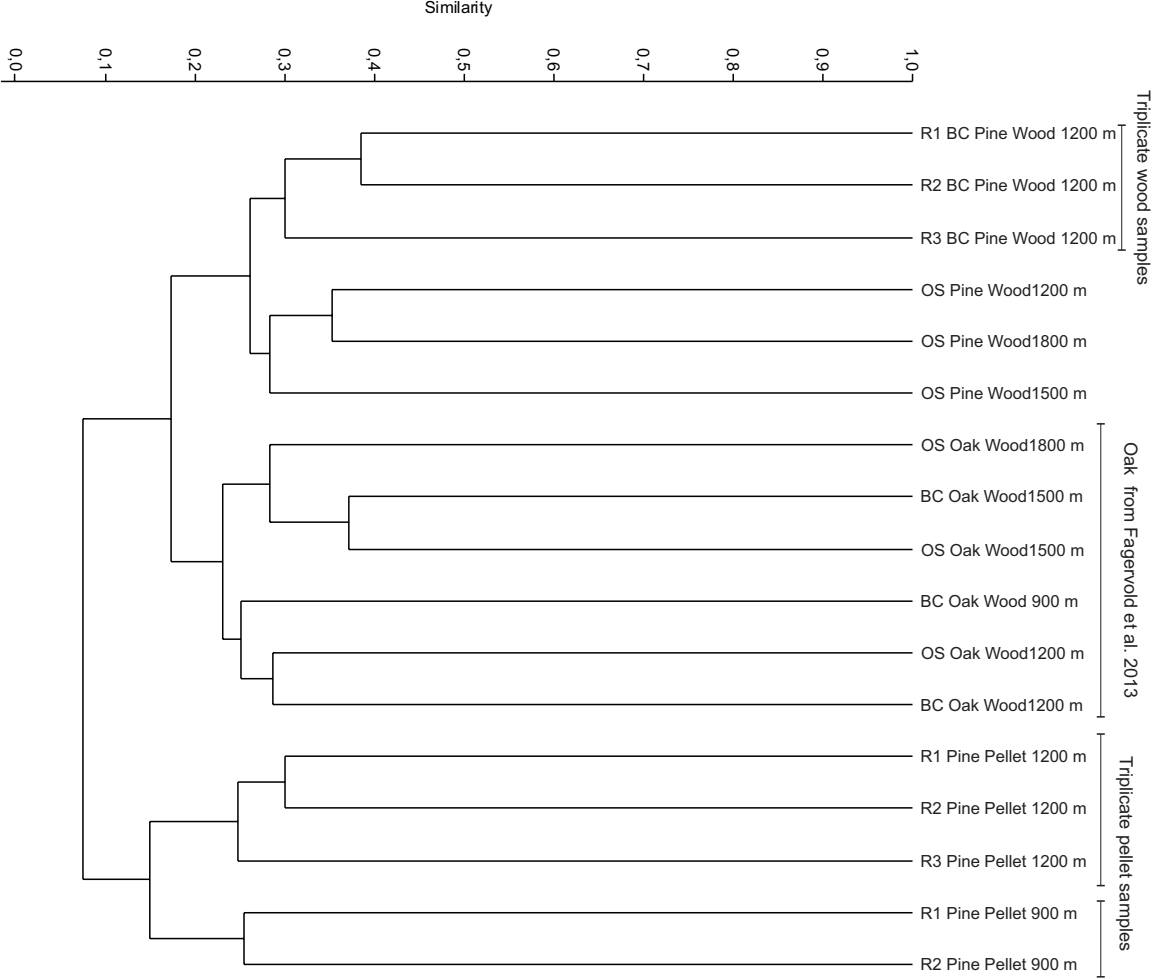

Supplement: Figure S1 — Clustering of all samples. Hierarchical clustering using the Bray Curtis index showing the similarity of the microbial communities between the different samples. (PDF) [file pone.0096248.s001.pdf]

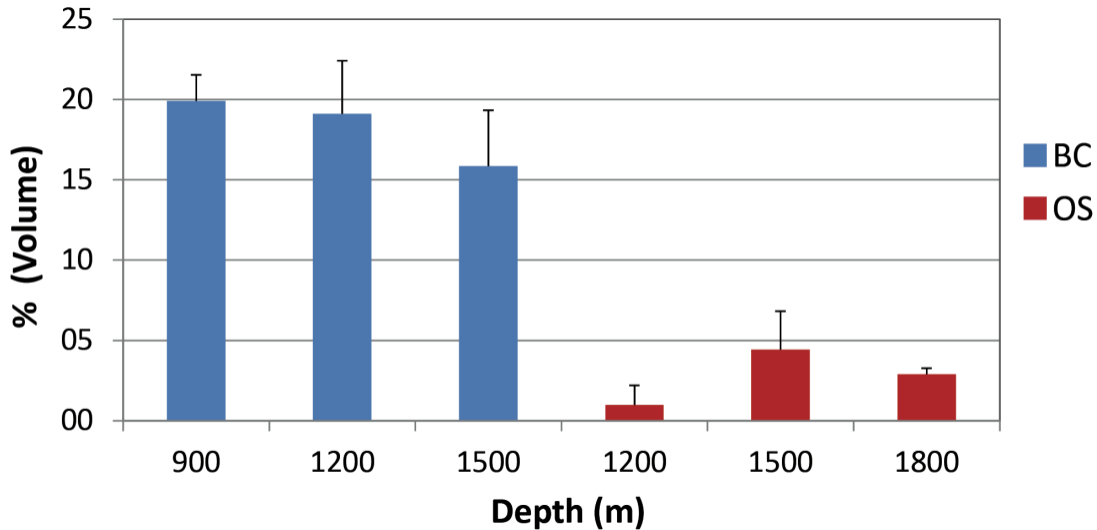

Supplement: Figure S2 — Wood consumtion. Percentage of consumed pine wood after immersion at various depths in Blanes Canyon (BC) and its adjacent open slope (OS), expressed as percentage of the initial volume of the respective wood cubes. (PDF) [file pone.0096248.s002.pdf]

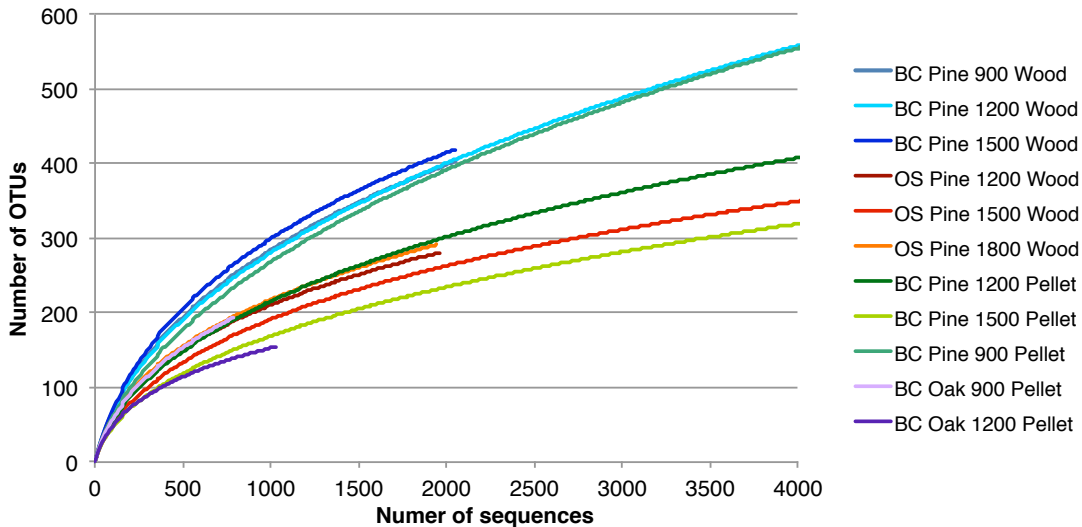

Supplement: Figure S3 — Rarefaction curve. Number of sequences versus OTUs formed for all sequenced samples, except the gill samples. Broadly, blue lines represents BC Pine Wood, redish lines OS Pine Wood, greenish lines Pine Pellet sampels and purple lines oak pellets. (PDF) [file pone.0096248.s003.pdf]
